# Advanced Sonographic Techniques in the Comprehensive Evaluation and Diagnosis of Male Infertility

**DOI:** 10.7759/cureus.62848

**Published:** 2024-06-21

**Authors:** Bharath Jain, Mahalakshmi Gaddi, Naveenkumar Nallathambi, Amisha Palande, Prashanth A, Gajalakshmi S, Prashannalakshmi A, B Holebasu, Roshan Prasad, Gaurav Mittal

**Affiliations:** 1 Radiology, S. S. Institute of Medical Sciences and Research Centre, Davanagere, IND; 2 Obstetrics and Gynaecology, SDM College of Medical Sciences and Hospital, Dharwad, IND; 3 Internal Medicine, Madras Medical College, Chennai, IND; 4 Physiology, Terna Medical College, Mumbai, IND; 5 Physiology, Mahatma Gandhi Institute of Medical Sciences, Wardha, IND; 6 Community Health, Veer Chandra Singh Garhwali Government Institute of Medical Science and Research, Srinagar, Dehradun, IND; 7 Obstetrics and Gynaecology, Tashkent Medical Academy, Urgench, Urgench, UZB; 8 Radiodiagnosis, KLE Jagadguru Gangadhar Mahaswamigalu Moorsavirmath Medical College and Hospital, Hubballi, IND; 9 Medicine and Surgery, Jawaharlal Nehru Medical College, Datta Meghe Institute of Higher Education and Research, Wardha, IND; 10 Internal Medicine, Mahatma Gandhi Institute of Medical Sciences, Wardha, IND; 11 Research and Development, Students Network Organization, Mumbai, IND

**Keywords:** varicocele, clinical laboratory techniques, physical examination, infertility, ultrasonography

## Abstract

Background

Infertility affects many couples, with male factors being responsible for over half of the cases. Male infertility can arise from various testicular illnesses, such as varicocele and cryptorchidism, as well as posttesticular disorders, like ejaculation abnormalities. Infertility is defined as the inability to conceive after 12 months of unprotected sexual activity or after six months for women over 35. Diagnostic techniques such as semen analysis and scrotal ultrasonography are done to evaluate conditions like varicocele and epididymo-orchitis. This study aims to assess the diagnostic utility of ultrasonography for male infertility and compare its findings with those from surgery and clinical care.

Methodology

All patients were referred to the Department of Radiology, Tertiary Care Hospital, South India, for transrectal and scrotal ultrasonography, using a high-frequency transducer with a frequency of 7.5 MHz and a color Doppler when necessary. The study included all male patients with infertility and abnormal semen analysis, as well as those with infertility accompanied by scrotal abnormalities detected during clinical examination. Patients were placed in the left lateral decubitus position for the transrectal ultrasonography examination. The testes and epididymis were thoroughly examined on both sides and compared regarding symmetry, size, texture, and vascularity.

Results

Varicocele was the most frequent anomaly detected by both clinical examination and ultrasonography. Ultrasound detected 30 cases of varicocele, whereas clinical examination diagnosed 15 cases. Hydrocele was identified in eight cases through clinical examination and in 15 cases through ultrasound. Epididymal cysts were found in five cases via clinical examination, while both clinical examination and ultrasonography discovered epididymitis in 10 cases. Overall, the number of anomalies detected by ultrasound was significantly higher than those found by physical examination, with a statistically significant p value of 0.001.

Conclusion

Transrectal ultrasound provides high-resolution imaging of the prostate, seminal vesicles, and distal vas deferens, which aids in diagnosing obstructive azoospermia. Imaging is a valuable supplement to clinical examination and laboratory studies for accurately identifying anatomy and abnormalities. Both transrectal and scrotal ultrasonography offer crucial information in diagnosing male infertility. Ultrasonography is more effective in identifying pathological abnormalities than clinical palpation.

## Introduction

Infertility poses a significant challenge for many couples, with male factors accounting for up to half of all cases. It is defined as the inability to conceive after a year of unprotected sexual intercourse. The importance of researching male infertility has grown due to its potential to identify treatable causes [1]. Imaging is crucial in diagnosing obstructive azoospermia and other structural issues, such as incorrect testicular positioning [2].

A multicenter study conducted by the World Health Organization (WHO) between 1982 and 1985 found that male factors accounted for 20% of infertility cases, female factors for 38%, both partners for 27%, and unexplained factors for 15%. In India, male factors contribute to over 23% of infertility cases among couples seeking treatment, with nearly half of all infertility cases linked to male reproductive disorders [3].

Male infertility can result from pretesticular, testicular, and posttesticular factors. Testicular causes include varicocele, cryptorchidism, and exposure to gonadotoxins; posttesticular causes include obstructions, ejaculation issues, and erectile dysfunction [1]. Pretesticular causes encompass inherited and acquired endocrinopathies, as well as conditions affecting follicle-stimulating hormone, gonadotropin-releasing hormone, luteinizing hormone, and androgen production.

Nonobstructive causes of male infertility include varicocele, endocrinopathy, chromosomal abnormalities, cryptorchidism, anabolic steroid use, exposure to gonadotoxins, primary testicular failure, and ejaculatory disorders. Obstructive causes include prostatic cysts, ejaculatory duct obstruction, and congenital bilateral absence of the vas deferens [4].

Transrectal ultrasound is essential for diagnosing obstructive azoospermia, providing high-resolution images of the prostate, seminal vesicles, and distal vas deferens. This condition is caused by anomalies in the ejaculatory duct, vas deferens, or epididymis, or by obstructions in sperm transport [1]. Scrotal ultrasonography examines the testicles, epididymis, and proximal vas deferens to detect abnormalities in the testis and paratesticular structures, such as varicoceles and epididymal issues, as well as secondary changes due to distal genital duct obstructions [1].

This study aims to demonstrate the value of imaging in diagnosing male infertility by examining the roles of transrectal and scrotal ultrasonography. It compares the ultrasound findings with clinical and surgical outcomes to highlight the diagnostic utility of these imaging modalities.

## Materials and methods

Study design and setting

This prospective cross-sectional study was conducted over the course of a year at the Department of Radiology, Tertiary Care Hospital, South India, involving 70 male patients presenting with infertility. Each patient underwent transrectal and scrotal ultrasonography, employing a high-frequency transducer set at 7.5 MHz, with color Doppler utilized as necessary.

Selection Criteria

The study excluded cases of infertility attributed to known female factors, impotence, and pretesticular causes. Inclusion criteria encompassed all male patients with abnormal semen analysis and those whose infertility was accompanied by concurrent scrotal abnormalities identified during clinical examination.

Data sources and variables

Transrectal ultrasonography was meticulously performed with patients positioned in the left lateral decubitus position, ensuring that a male chaperone was present throughout the procedure to provide comfort and maintain protocol standards. The importance of patient comfort and compliance was emphasized, with detailed explanations provided before and during the procedure. Scrotal ultrasound scans were conducted using a Mindray DC-8 diagnostic ultrasound machine equipped with a high-resolution 7.5-MHz transducer, which is essential for obtaining detailed images of the scrotal contents.

Before the examination, patients were thoroughly briefed on the procedure, including the purpose and steps involved, to alleviate any anxiety and ensure cooperation. They were positioned supine on the examination table, with their pants and underwear adjusted halfway up their thighs to expose the scrotal area adequately. A folded towel was placed between the patient's knees to elevate and support the scrotum, facilitating optimal imaging conditions. The penis was positioned over another towel placed over the suprapubic area to prevent interference with the scanning process.

Coupling gel, essential for eliminating air gaps between the skin and the transducer, was liberally applied to both scrotal sacs. This gel enhances the transmission of ultrasound waves, ensuring clear and precise images. The testicles and epididymis were then thoroughly examined in both longitudinal and transverse planes. This comprehensive examination allowed for a detailed evaluation of the scrotal contents, focusing on assessing symmetry, size, texture, and vascularity. These parameters are critical for identifying abnormalities such as varicoceles, hydroceles, epididymal cysts, and other pathological conditions.

Comprehensive patient histories were meticulously documented, encompassing detailed physical examinations, laboratory investigation findings, and ultrasound results. These histories included information on previous medical conditions, family history of infertility, lifestyle factors, and any prior treatments or surgeries related to infertility. Integrating these histories with the ultrasound findings provided a holistic view of each patient's condition, facilitating accurate diagnosis and effective treatment planning.

Statistical analysis

Data were compiled into a master graphic using Microsoft Excel (Redmond, WA) and analyzed with SPSS version 20 (IBM Corporation, Armonk, NY). Descriptive statistics were employed to characterize categorical and continuous variables, utilizing frequency analysis, percentage analysis, and mean analysis. Statistical information was presented as mean ± standard deviation (SD). The association between two qualitative variables was determined using the chi-square test.

## Results

Table 1 presents the age distribution of infertile men along with the mean and SD of their ages. Among the participants, the majority fell within the age range of 26-30 years, comprising 57.14% of the total cases. Those aged 21-25 constituted 21.42%, while individuals aged 31-35 and 36-40 years accounted for 11.43% and 10%, respectively. In total, 70 participants were included in the study. The participants' mean age was 28.50 years, with an SD of 3.88 years, indicating the variability in age within the sample population. Most cases fell within the sperm count range of 5-10 million per milliliter, totaling 22 instances. Additionally, there were 20 cases diagnosed with azoospermia.

**Table 1:** Age distribution of infertile men with mean and SD n: frequency of cases; %: percentage of cases; SD: standard deviation

Age range (years)	Frequency (n)	Percentage (%)
21-25	15	21.42%
26-30	40	57.14%
31-35	8	11.43%
36-40	7	10%
Total	70	100%
Mean ± SD	28.50 ± 3.88	

Table 2 presents the findings from the physical examination conducted on infertile men. The examination identified various conditions, with varicocele being the most prevalent, observed in 80% of cases on the left side, 13.33% on the right side, and 6.67% bilaterally, totaling 15 cases. Hydrocele was also noted, with 37.5% occurring on the left side, 37.5% on the right side, and 25% bilaterally, totaling eight cases. Additionally, other findings included epididymitis in 47.62% of cases, nonpalpable vas deferens in 9.52% of cases, single left and right testes in 9.52% of cases each, and epididymal cysts in 23.81% of cases, summing up to 21 cases in total.

**Table 2:** Results from physical examination n: frequency of cases; %: percentage of cases

Findings		Frequency of cases (n)	Percentage (%)
Varicocele	Left	12	80%
	Right	2	13.33%
	Bilateral	1	6.67%
	Total	15	100%
Hydrocele	Left	3	37.5%
	Right	3	37.5%
	Bilateral	2	25%
	Total	8	100%
Others	Epididymitis	10	47.62%
	Nonpalpable vas deferens	2	9.52%
	Single left testis	2	9.52%
	Single right testis	2	9.52%
	Epididymal cyst	5	23.81%
	Total	21	100%

Table 3 summarizes the results of scrotal ultrasound examinations. Varicocele was most prevalent, with 66.67% on the left, 10% on the right, and 23.33% bilaterally, totaling 30 cases. Hydrocele occurred in 40% on both sides and 20% bilaterally, totaling 15 cases. Epididymal cysts were observed in 66.67% on the left, 6.67% on the right, and 26.67% bilaterally, totaling 15 cases. Epididymitis affected 40% on each side and 20% bilaterally, summing up to five cases. Other findings included testicular abnormalities, found in 50% on the left, 33.33% on the right, and 16.67% bilaterally, totaling six cases.

**Table 3:** Scrotal ultrasound findings n: frequency of cases; %: percentage of cases

Findings		Frequency of cases (n)	Percentage (%)
Varicocele	Left	20	66.67 %
	Right	3	10%
	Bilateral	7	23.33%
	Total	30	100%
Hydrocele	Left	6	40%
	Right	6	40 %
	Bilateral	3	20%
	Total	15	100%
Epididymal cyst	Left	10	66.67%
	Right	1	6.67%
	Bilateral	4	26.67%
	Total	15	100%
Epididymitis	Left	2	40%
	Right	2	40%
	Bilateral	1	20%
	Total	5	100%
Others	Left	3	50%
	Right	2	33.33%
	Bilateral	1	16.67%
	Total	6	100%

Table 4 presents the association between physical examination findings and ultrasound results. Varicocele was identified in 15 cases during physical examination, whereas ultrasound detected it in 30 cases. A physical examination identified eight cases of hydrocele, compared to 15 cases detected by ultrasound. Similarly, epididymal cysts were found in five cases through physical examination and 15 cases via ultrasound. Epididymitis was observed in 10 cases through physical examination and five cases through ultrasound. The chi-square test yielded a value of 17.10, indicating a statistically significant association (p < 0.001) between physical examination and ultrasound findings.

**Table 4:** Association between physical examination and scrotal ultrasound findings χ2: chi-square P value <0.01 is considered to be statistically significant

Findings	Physical examination		Scrotal ultrasound findings
Varicocele	15		30
Hydrocele	8		15
Epididymal cyst	5		15
Epididymitis	10		5
Total	38		65
χ2		17.10	
P value		<0.001	

Table 5 summarizes the transrectal ultrasound findings. Among the diagnostic findings, four cases showed an absent vas deferens. Additionally, there were two cases of midline prostatic cysts confirmed during surgery and two cases of seminal ductal ectasia.

**Table 5:** Transrectal ultrasound findings n: frequency of cases

Diagnostic finding	Frequency of cases (n)
Absent vas deferens	4
Midline prostatic cyst	2 (confirmed on surgery)
Seminal ductal ectasia	2

## Discussion

Many men who were previously considered infertile are now fathers, thanks to the rapid development of breakthrough management techniques for male factor infertility over the past decade. Male factors are responsible for half of all infertility cases. Early identification and treatment of male infertility heavily rely on scrotal and transrectal ultrasonography. In certain situations, addressing the underlying cause of ductal system obstruction can restore the patient's fertility.

The goal of the current study, which involved 70 infertile men at the Department of Radiology, Tertiary Care Hospital, South India, was to determine the significance of scrotal and transrectal ultrasonography in male infertility. Clinical and surgical findings were compared with radiological findings.

The study conducted by Brunereau et al. [5] found that the mean age of the patients was 29.20 years with a range of 24-40 years, which was comparable to the mean age of 28.50 ± 3.88 years in the current study. The age range recorded by Pethiyagoda AUB and Pethiyagoda K [6] was 25-40 years old, with a mean age of 35.15 years. This illustrates shifting societal socioeconomic trends, especially in wealthy nations where marriage and childbearing are increasingly postponed. The research conducted by Chauhan and Jha [7] and Mohi et al. [8] produced similar results.

In accordance with WHO recommendations, patients with sperm counts of less than 20 M/mL were included in the current study. The study group comprised 20 patients with azoospermia and 22 with sperm counts between 5 and 10 M/mL. Goullet et al. [9] examined 609 infertile males, with 191 having azoospermia and 418 having oligoasthenoteratospermia. The study by Moon et al. [10] involved 20 azoospermic infertile men. Qublan et al. [11] studied 234 infertile men with sperm counts below 10 M/mL. The semen parameters of this study align with those reported by Chauhan and Jha [7], Mohi et al. [8], and Brunereau et al. [5].

In the current investigation, varicocele was the most frequently found anomaly on palpation, consistent with findings by Mohi et al. [8] and Chauhan and Jha [7]. Varicocele was found in 15 (21.43%) cases during physical examination. In their study of 262 infertile males, Eskew et al. [12] found clinically palpable varicocele in 89 (34%) patients, while ultrasonography detected it in 168 (64%) patients. Preutthipan and Nicholas [13] reported left-sided varicocele in 32 patients, right-sided varicocele in two patients, and bilateral varicocele in 6 out of 110 patients.

In this study, the incidence of various pathological findings on scrotal ultrasound included varicoceles (42.86%), hydrocele (21.43%), epididymal cysts (21.43%), epididymitis (7.14%), cryptorchidism (5%), testicular microlithiasis (5%), and bilateral testicular cysts (3%). With the exception of five patients-three with hydrocele and two with cryptorchidism-all testes were normal in size, shape, and echo pattern, with no focal lesions. Gordon et al. [14] found similar results in their study of 53 infertile males, including varicoceles (34%), testicular microlithiasis (8%), and a few cases of testicular tumors and thickened epididymis. Jequier et al. [15] reported varicocele (52%), epididymal cysts (22%), testicular microlithiasis (6%), hydrocele (4%), and testicular cancer (0.4%) among 1,203 infertile men. Ibrahim et al. [16] observed varicocele (39%), hydrocele (26%), epididymo-orchitis (7%), epididymal cysts (5%), and testicular microlithiasis (4%) in 115 infertile males.

Clinical palpation in the current study found left varicoceles in 12 patients, bilateral varicoceles in one patient, and right varicoceles in two patients. Ultrasound detected left varicoceles in 20 patients, bilateral varicoceles in seven patients, and right varicoceles in three patients. Dogra et al. [17] reported that 29% of 1,372 infertile males had varicocele detectable by ultrasonography, with 60% of these cases being clinically palpable. They noted that color Doppler ultrasound has nearly 100% sensitivity and specificity for detecting varicocele. Sakamoto et al. [18] found varicocele in 45% of 545 infertile males during physical examination and in 51.4% using ultrasonography.

Transrectal ultrasonography in the present study found dilated seminal vesicles in two cases (2.86%), absent vas deferens in four cases (5.71%), and midline prostatic cysts in two cases (2.86%). Ho et al. [19] reported congenital absence of the vas deferens in 8% of 387 patients. The current study's results are similar to those reported by Abdulwahed et al. [20]. The relationship between physical examination and ultrasound findings was statistically significant, with a p value of less than 0.001. These results are consistent with studies by Chauhan and Jha [7] and Mohi et al. [8], which also demonstrated a strong association between clinical and ultrasonographic findings in infertile men.

Limitations of the study

The study has some limitations, including a small sample size of 70 patients and being conducted at a single center, which limits the generalizability of the findings. The study lacks longitudinal follow-up to assess long-term outcomes postdiagnosis and treatment. Reliance on clinical palpation, which can be subjective, may affect the accuracy of initial findings compared to ultrasonography. Additionally, the study did not compare the effectiveness of different treatment modalities or include a broad age range, potentially overlooking age-related factors.

## Conclusions

Ultrasonography is significantly more effective than clinical palpation in identifying pathological abnormalities. While clinical palpation is useful, it often lacks the precision and detail that ultrasonography provides. Ultrasonography can detect subtle changes and abnormalities within the reproductive organs, such as cysts, ductal ectasia, or absent vas deferens, which might not be detectable through palpation alone. This superior diagnostic capability ensures a more comprehensive patient evaluation, leading to better informed decisions regarding their treatment options.
